# Use of a Smartphone-Based Augmented Reality Video Conference App to Remotely Guide a Point of Care Ultrasound Examination

**DOI:** 10.3390/diagnostics9040159

**Published:** 2019-10-24

**Authors:** Davinder Ramsingh, Cori Van Gorkom, Matthew Holsclaw, Scott Nelson, Martin De La Huerta, Julian Hinson, Emilie Selleck

**Affiliations:** 1 Department of Anesthesiology, Loma Linda University Health, 11234 Anderson Street MC-2532, Loma Linda, CA 92354, USA; CVangorkom@llu.edu (C.V.G.); mholsclaw@llu.edu (M.H.); jhinson@llu.edu (J.H.); ESelleck@llu.edu (E.S.); 2 Department of Orthopedic Surgery, Haiti Adventist Hospital, Route De La Mairie De Carrefour, Diquini 63, Haiti; scnelson@llu.edu (S.N.); Delahuertam@yahoo.com (M.D.L.H.)

**Keywords:** point of care ultrasound, augmented reality, telemedicine

## Abstract

Reports on the use of various smartphone-based video conference applications to guide point-of-care ultrasound (POCUS) examinations in resource-limited settings have been described. However, the use of an augmented reality-enabled smartphone video conference application in this same manner has not been described. Presented is a case in which such as application was used to remotely guide a point of care ultrasound examination.

## 1. Introduction

The ability to improve the quality of care to resource-limited settings is often a logistical challenge resulting from the lack of specialized medical practitioners and services. This is often secondary to geographic, demographic, and socioeconomic factors. The implementation of technology in healthcare is often a contributor to this problem rather than a solution. However, recent innovations in smartphone technology and point-of-care ultrasound (POCUS) devices have proven to be key examples of how technological advances are poised to elevate the quality of care in resource-limited settings. Indeed, the use of smartphone devices to provide real-time video conferences has proven to improve rural medicine across many medical specialties [1,2,3,4,5]. Similarly, the advancements in POCUS technology have greatly facilitated the ability to perform ultrasound exams in remote patient care settings.

Point-of-care ultrasound refers to the use of ultrasonography at the patient’s bedside for diagnostic and therapeutic purposes [6]. The provider acquires and interprets all images in real-time and then uses that information to diagnose and direct therapies. Of note, POCUS has been identified as the most rapidly growing sector in medical ultrasound imaging [7]. Recent advances in this technology include improved image quality as well as a significant reduction in price, with handheld devices costing approximately 1/20th the price of devices ten years ago (from USD 40,000+ to USD 2000).

Recently, smartphone-based video conference technologies have been used with point-of-care ultrasound. Several studies have demonstrated the ability to remotely educate, guide, and provide image interpretation of POCUS examinations. While these studies have shown promise, a new modality, augmented reality (AR), has recently been implemented in the point-of-care ultrasound education space [8]. The use of AR has demonstrated utility in the remote guidance of POCUS [8]. However, the use of AR to improve remote medical training has mostly been described with the use of specialized equipment that may not be readily available in resource-limited environments.

**Figure 1:**
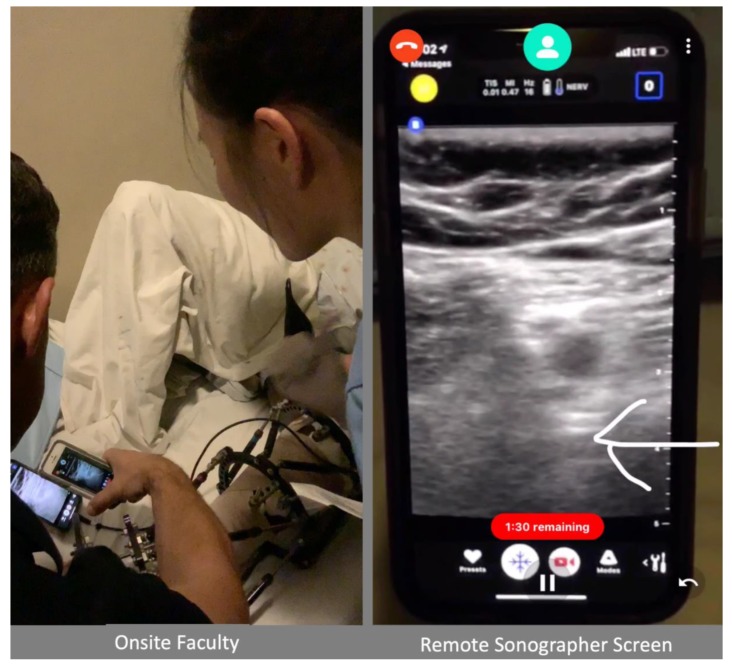
Overview of Onsite and Remote Augmented Reality Enhanced Video Communication. The white arrow indicates the femur, which was identified for anatomy review during remote guidance communication.

This report highlights the use of a novel smartphone application (Vuforia Chalk, San Diego CA, USA) to provide AR remote assistance to guide a POCUS examination. The application works on most smartphone devices and web browsers and provides an augmented reality video conference interface that allows each user to notate the other’s environment (see Figure 1). The ease of use and widespread applicability across multiple smartphone platforms allows this program to potentially improve the availability of remote AR guidance to teach POCUS in resource-limited settings. This non-sponsored case report was a proof-of-concept evaluation on the feasibility of using this AR application to improve remote guidance of POCUS examinations in a resource-limited environment. Specialty-trained providers from a tertiary care center, Loma Linda University Medical Center (LLUMC), in California, USA, successfully used this application to provide remote AR guidance for a POCUS examination at a rural hospital in Port-au-Prince, Haiti.

## 2. Description of the Case

Faculty from the tertiary care center traveled with a low-cost ($2000 USD) handheld portable ultrasound device (Butterfly Network, Guilford, CT. USA) and a *Chalk*-enabled smartphone (iPhone 8, Apple Cupertino, CA. USA) to the Hôpital Adventiste d’Haiti. During the visit, the onsite and visiting faculty identified a 35-year-old male patient scheduled to undergo an external fixator removal and replacement. The patient required a regional anesthesia popliteal nerve block. The ability to use ultrasound to perform this block was not routinely available, and local providers had not been trained to perform this procedure. To evaluate the capability of the AR application to provide remote guidance for this procedure a connection was established, via the *Chalk* application, between the visiting faculty’s smartphone in Port-au-Prince, Haiti to a remote faculty in CA, USA. Consent was obtained from the patient to report this case.

The connection was established over a mobile 4g hotspot via an iPhone 5 (Apple Cupertino, CA. USA) provided by the onsite faculty. *Chalk* was used to send a call to the expert ultrasonographer (San Diego, CA, USA), who then used the AR platform to guide probe placement on the patient to obtain the appropriate ultrasound image. Once the onsite examiner had obtained the appropriate probe position and ultrasound image, the smartphone camera was adjusted to visualize the ultrasound image from the POCUS device. Specifically, the smartphone POCUS exam was placed on the patient’s bed in-between their legs and the second smartphone with the AR platform was held over this device such that the ultrasound image and the AR notations could be visualized by the physician performing the exam (Figure 1). Importantly the user holding the smartphone with the AR platform was not the same person performing the procedure and would adjust the smartphone position to allow appropriate visualization for the proceduralist. The remote expert then highlighted relevant anatomy and identified the nerve on the ultrasound image via the AR platform.

The procedure was performed successfully, and the nerve block demonstrated appropriate efficacy. After the procedure, the one onsite and the one remote provider completed a survey on the image quality of the video connection. In addition, the remote provider completed a survey of the image quality of the ultrasound image viewed from the video conference app and the onsite provider completed a survey of the AR notations created by the remote provider during the guidance of the nerve block. All surveys were scored using a validated 5-point Likert scale [9].

Survey results showed that the image quality of the video communication was rated 5/5 for both video communication and ultrasound image interpretation by the one onsite and one remote practitioner. The onsite practitioner scored the clarity of the AR notations to identify probe placement position on the body as 5/5 and identification of anatomy and nerve on the ultrasound image as 4/5.

## 3. Discussion

Technologic advances in medical ultrasound imaging are helping remove the barriers of costs and portability. These innovations are improving the ability to use medical ultrasound in resource-limited settings as a point of care device. However, a barrier that remains is the skill/training of the providers in these settings. Programs to teach POCUS, in-person, have demonstrated to be effective [10], but have a high cost and can be difficult to repeat/grow. The use of real-time video conferencing has demonstrated to be effective for remote POCUS guidance and training [11,12]. The application of such a remote guidance and telecommunication system has demonstrated a positive clinical impact. Kolbe et al. reported a change in management in 48% of patients in a rural village in Nicaragua after the implementation of a remote guidance and telecommunication system between expert sonographers around the world and local practitioners [13]. The application of smartphone-based video conference platforms also has proven clinical utility [12]. Robertson et al. demonstrated successful communication between intensivists at a tertiary care center and non-physician health care providers in a low-income country, which demonstrated successful ability to both educate POCUS image acquisition techniques as well as allow for appropriate image quality for remote clinical interpretation [14].

In recent years, the development of AR has also been applied to POCUS. Wang et al. evaluated the feasibility of using a specific AR hardware/software platform, Microsoft HoloLens (Redmond, WA, USA), to remotely guide novice medical trainers through a trauma ultrasound examination [8]. While this does offer a tremendous opportunity to expand POCUS education, it may be less impactful in resource-limited environments. Advances in smartphone applications now allow for the use of real-time AR enhanced video communication without the need for expensive hardware. This potentially has broad implications in resource-limited areas by improving the ability to provide remote POCUS education/guidance with low-cost smartphone and POCUS devices. While a limited example, this case demonstrates how these devices can be implemented to provide improved bedside assessment and therapies in a resource-limited environment.

Our report demonstrates the use of a smartphone app that allows for the creation of a real-time augmented reality environment in a manner very similar to common smartphone video conference applications. This proof-of-concept case report presents positive feedback from all of the providers involved and supports further exploration in this area. Of note, none of the onsite physicians involved in the case had used the Chalk app before this event. Additional discussion after the event between the onsite and remote providers highlighted that the use of this platform was an improvement over traditional smartphone video conferencing by allowing both users to provide real-time visual cues over the ultrasound image during the procedure. In addition, the AR interface provided a greater ability for depth perception compared to traditional smartphone video conferencing.

Importantly, there are several limitations for the setup described in this report. While providers reported no issues with screen glare, the use of two mobile devices to achieve the AR guidance resulted in a limitation of the field-of-view of the remote examiner. In addition, the setup described requires another individual to hold and manipulate the AR smartphone. Additionally, the placement of the ultrasound and AR devices will be different based on patient position and care setting. Finally, the AR video communication in this case report did not include the transfer of any protected health information, as this requires secure communication pathways, which have previously been described [15]. Truly, applications such as the one described in this report would require the development of these securities to provide the maximum benefit. Future evaluations of these technologies should seek to address each of these items.

Indeed, the potential widespread availability of a smartphone-based augmented reality training/guidance application makes this platform very exciting for improving healthcare in resource-limited environments by potentially providing a higher level of communication than standard video conferencing. It is the authors’ hope that this report can be an example to stimulate formal research in this area.
